# Diet and Disease Development: Present and Future

**DOI:** 10.3390/nu16213681

**Published:** 2024-10-29

**Authors:** Sonia Saad

**Affiliations:** Department of Medicine, Kolling Institute of Medical Research, University of Sydney, Sydney 2065, Australia; sonia.saad@sydney.edu.au

The present Editorial provides an overview of the Special Issue “Diet and Disease Development” recently published in *Nutrients*.

The relationship between diet and disease development or future programming in certain diseases is well known [1,2,3]. Diet is the key factor influencing the composition of the gut microbiota and an important factor in the microbiome balance [4]. Alterations in the microbiome balance play an important role in physiology and disease states [5]. Hence, recent research has focused on using natural therapies or dietary supplements to modulate the microbiota for disease prevention or to better manage disease development. The mechanisms involved in the direct and transgenerational effects of bad dietary choices on disease development are not clear. It is also not clear, to date, whether alterations in the microbiota can affect disease onsets or limit disease progression, or whether using dietary supplements or microbiota modulators can limit disease or prevent future disease development.

This Special Issue includes 14 literature reviews that describe the role of diet in disease development and future disease predisposition. Through this Special Issue, we understand the role of microbiota dysbiosis in disease development and the therapeutic effect of nutrients on different diseases such as constipation, menopausal health management, cognitive health, traumatic brain injury, dementia, cystic fibrosis, diabetes, stroke, cardiovascular disease and pancreatic disease. We also understand the importance of diet modification before or during pregnancy on maternal and offspring’s health outcomes and whether the microbiota profile changes in obese individuals after bariatric surgery. The therapeutic potential of faecal transplantation and nano-delivery are also discussed.

Briefly, Pan et al. described the crosstalk between the gut microbiome and colonic motility in chronic constipation and examined the potential mechanisms and effect of microbiota modulation [6]. The authors summarised the effect of known microbial metabolites on gastrointestinal physiology and described the role of the enteric nervous system as well as the mechanisms whereby the gut microbiome can affect the central nervous system, the immune system, intestinal epithelial barrier function, intestinal secretion function and sex hormones. The role of probiotics in these mechanisms was also examined and randomised controlled and parallel-group trials involving micro-ecological intervention in children and adults with constipation were summarised to examine the clinical application of probiotics in constipation.

Chen et al. on the other hand examined the role of lactic acid bacteria and whether they can be used for menopausal health management [7]. This review described both menopausal symptoms and the mainstream method to manage menopause. The potential therapeutic role of lactic acid and the mechanisms of effects were well summarised. The role of lactic acid on oxidative stress, oestrogen production, osteoporosis, dyslipidaemia, obesity, the vaginal microbiome and the central nervous system were also discussed, in addition to current gaps. Shrestha et al. summarised the effect of essential fatty acids such as linoleic acid and alpha-linolenic acid, omega 6 and omega-3 polyunsaturated fatty acids, respectively, in cystic fibrosis disease progression [8]. The roles of the patient’s genotype in the symptoms of cystic fibrosis and essential fatty acid levels were also reported, in addition to the sex differences and phenotypic variety. Campbell J wrote a comprehensive review about the role of NAD+ supplementation as well as its precursors in cognitive ability [9]. The role of NAD+ precursors in dementia, diabetes, stroke and traumatic brain injury, as well as in healthy individuals, was summarised. In addition, the author described completed and ongoing clinical trials assessing the potential therapeutic effects of NAD+ precursor supplementation and their findings to date. Li et al. described the effect of urolithin A on pancreatic diseases [10]. Urolithin is a dietary metabolite of the intestinal microbiota that is produced following the ingestion of the plant-based food ingredients ellagitannins and ellagic acid, found in various berries, nuts and seeds, for example. The pharmacokinetics of urolithin A was described as well as its biological activity including its known effects on inflammation, oxidative stress, aging, metabolic homeostasis, Alzheimer’s disease and cognitive impairment. The potential effect on pancreatic diseases such as pancreatitis and pancreatic cancer, as well as on pancreatic tissues and function, was summarised and the potential mechanisms of this function were discussed. Dong et al. examined the potential roles of dietary anthocyanins, a polyphenol pigment and secondary metabolite of plant-based food that is widely present in fruits and vegetables, in inhibiting vascular endothelial cell senescence and preventing cardiovascular diseases [11]. The metabolism and activities of anthocyanins as well as their structure are described, and their physiological activities in cancer, inflammation, oxidative stress defence, liver disease, blood sugar levels, aging and metabolic disease are summarised. In addition, anthocyanin bioavailability and interaction with the gut microbiome and the complex mechanisms in cardiovascular health are discussed. Pei et al. reviewed the potential therapeutic effects of pearl millet in glycaemic control and diabetes mellitus [12]. The nutritional significance of pearl millet in comparison to other millets was described and the general biomedical application in difference diseases was also covered. The review stressed the importance of conventional wholegrain and multigrain alternatives to carbohydrates and the health benefits and biomedical applications.

Furthermore, Liu et al. described the metabolic homeostasis of amino acids and their role in diabetic kidney disease [13]. Through this review, the authors described the importance of amino acids as a dietary approach for the management of diabetic kidney disease and how amino acid homeostasis can contribute to the renal hemodynamic response and the alteration of glomerular filtration in diabetic patients. The role of different types of amino acids and the potential effect on diabetic kidney disease progression were also summarised. The authors additionally offered an optimized protein diet strategy while managing amino acid proportion and homeostasis to reduce the risk of diabetic kidney disease. Fotheringham et al. described the role of advanced glycation end products (AGEs) in chronic kidney disease and examined whether the modern diet ages the kidney [14]. The pathways that drive AGE formation and regulation within the body are summarised as well as the evidence for dietary AGEs and endogenously produced AGEs in driving pathogenesis in diabetic and non-diabetic kidney disease. The potential therapeutic effect of targeting AGEs in kidney disease was discussed. The review summarised the effect of the Western diet and its risk to kidney disease and described the factors that regulate AGE turnover in the body. In addition, the role of AGE receptors is described as well as the effect of AGE homeostasis imbalance on the development of diabetes and kidney disease. The role of dietary AGE intake and its effect on endogenous AGE production are also discussed. The role of AGE-lowering strategies in clinical studies is summarised and current gaps are identified.

The importance of maternal diet on offspring’s health outcomes is also described as well as the effect of diet modification on maternal and foetal health outcomes. Briefly, Witek et al. described the effect of high-sugar diet consumption, metabolism and health impacts particularly its effect on the development of substance use disorder [15]. The daily limit of carbohydrate was described as well as the detrimental effect of added sugar consumption. The mechanism of hormonal and neuronal regulation of sugar intake was also summarised as well as adverse effects related to increased sugar consumption. Preclinical studies related to the effect of a high-sugar diet on behaviour and neurochemical consequences were summarised. The current knowledge about the effect of maternal and postnatal sugar intake on the development of mental disorders in offspring was also covered. In addition, Rodrigo et al. examined the effect of diet modification before or during pregnancy on maternal and foetal outcomes [16]. Through this review, the authors described the known effects of maternal obesity on the mothers and offspring and the benefits of dietary intervention, whether before or during gestation on maternal and foetal health outcomes. The pathological pathways of disease development were elucidated from different preclinical models. The authors also discussed different methods to limit or reduce the impact of maternal obesity as well as the potential for these interventions to be translated to humans. The microbiota profile in obese individuals before and after bariatric or gastric sleeve surgery was described by Coimbra et al., who evaluated the changes in the gut microbiota in obese adults receiving bariatric surgery over the past ten years [17]. The abundance of different types of gut microbiota was determined in the same patients before and after a bariatric surgery and the alteration in the gut microbiome was also compared to patients receiving sleeve gastrectomy.

Furthermore, the therapeutic potential of faecal transplantation and nano-delivery as novel tools to deliver functional food or promote a healthy microbiota to improve health outcomes were also discussed. Bian et al. summarised the current evidence for the role of gut microbiota dysbiosis in chronic kidney disease from animal and human studies [18]. The authors also described the mechanisms of chronic kidney disease development and the potential therapeutic role of faecal microbiota transplantation in chronic kidney disease either alone or as an adjunctive therapy. The review summarised experimental and clinical studies related to the use of faecal transplantation in kidney disease and identified current knowledge gaps. In addition, Clarence et al. unravelled the therapeutic potential of the nano-delivery of functional foods in chronic respiratory diseases [19]. The authors wrote a comprehensive review about the use of functional foods in the management of inflammatory lung disease and summarised studies in which nanotechnology was used for compound delivery as well as clinical trials that were conducted to assess the effect of nanotechnology-based drug delivery containing beneficial functional foods for neurological disorders and lung diseases. The effectiveness and safety of such technology, as well as the limitations, were also discussed.

This Special Issue highlights reviews which contribute expert insights into the dietary nutrients affecting disease development at both the molecular and clinical levels. I hope it will of particular benefit to the readers of *Nutrients*.
